# DNA barcoding of Mobulid Ray Gill Rakers for Implementing CITES on Elasmobranch in China

**DOI:** 10.1038/srep37567

**Published:** 2016-11-23

**Authors:** Yan Zeng, Zhongze Wu, Chunguang Zhang, Zhibin Meng, Zhigang Jiang, Jie Zhang

**Affiliations:** 1 Institute of Zoology, Chinese Academy of Sciences, Beijing 100101, China; 2 Endangered Species Scientific Commission, People’s Republic of China, Beijing 100101, China; 3 CITES Management Authority, People’s Republic of China, Beijing, 100714, China

## Abstract

The Convention on International Trade in Endangered Species of Wild Fauna and Flora (CITES) has been counted on for conserving threatened marine fish since it regulates the commercial international trade of these species. Implementation of the international treaty for Mantas included on CITES Appendix II is challenging due to insufficient information on species identification and markets management. To fill the gap in such aspects, we identified five species of Mobulid rays (*Mobula* spps. and *Manta* spp) by using COI and NADH2 mtDNA markers in dried ray gill rakers from Chinese markets, namely, *Mobula japonica* (representing 54.8% of the sample set), *M. tarapacana* (14.4%), *M. kuhlii* (13.3%), *M. thurstoni* (6.4%), along with *Manta birostris* (11.2%; CITES Appendix II). The utilization and conservation statuses of these species were discussed. Based on combination of DNA barcodes and key morphological characters, we developed a three-step process for identifying the gill rakers of Mobulid rays which has been adopted by frontline enforcement in China. We hope that our work can serve as a foundation and basis to reinforce objectives of international treaties, regulation of consumer-driven markets, regional cooperation, and national fishery management on endangered elasmobranchs in China as well as related countries.

At its Sixteenth Meeting of the Conference of the Parties in March 2013 in Bangkok, the Convention on International Trade in Endangered Species of Wild Fauna and Flora (CITES) adopted a series of historic proposals, including listing seven species of commercially exploited elasmobranch fish under Appendix II of the Convention. The Appendix-II listings, based on the CITES treaty, means that although they are not necessarily now threatened with extinction, they may become so unless trade is strictly regulated; the listing aims to ensure that the international trade will not threaten the survival of the wild population of the species and to provide a method of tracing and monitoring their trade. The effective date of listings of sharks and rays under CITES Appendix II was delayed for 18 months until September 2014 to give governments time to resolve technical and administrative issues. Some of the challenges include scientific issues such as: limited knowledge for the identification of products in trade, such as processed shark fins, meat, cartilage and gill rakers; insufficient information about wild populations; lack of fishery information; and insufficient data and information on utilization, marketing and trade of sharks and rays^1^,^2^,^3^.

Among newly listed elasmobranchs, *Manta* rays are iconic species due to the largest size in rays and the significant rates of decline in population sizes in recent years^4^,^5^,^6^. According to recent reports, the increase in the trade in ray gill rakers in the Asian markets, especially in China, has posed a threat to the survival of *Manta* rays^2^,^5^,^6^,^7^,^8^,^9^,^10^. However, trading specimens of Manta ray gill rakers were always in mixed shipment with other Mobulid rays^9^, including two recognized *Manta* species and nine species of its sister genus, *Mobula*^6^,^11^,^12^,^13^. Despite China’s large population, the demand for gill plates, or Peng Yu Sai (the Chinese name for the Mobulid ray gill rakers), is not from the majority of the Chinese, but from the people of Hong Kong, Guangdong and Guangxi^14^,^15^.

Since CITES may be the only international treaty about biodiversity conservation that has legal mechanisms, it is considered to be an ideal complement to national and regional management of marine fish. CITES still lacks experience in fish management and in determining the relationship with other international agreements about marine fish^16^, however, the success of the implementation of the listing of Mantas depends on a well-designed strategy to address surveillance, compliance and enforcement. The substantial implementation of the international cooperation among the source country, the transit country, and the consumer country must be immediately started. In time the paper prepared, a proposal for the Convention of Parties of CITES was submitted to listing all species of Mobula into the Appendix II of CITES. One of reasons of the listing suggested that the specimens of *Mobula* spps. in trade, mainly the dried gill plates, were unlikely to be able to distinguish by enforcement officers^17^. Thus, some information such as user friendly guides of species identification for experts and non-experts are urgently needed^1^ and must be shared among all parties.

In the present study, we sampled at the mainland Chinese markets, adopted DNA barcoding methods for species identification and identified the species components of ray gill rakers. Moreover, the DNA identity of the gill rakers was compared to the morphology of the dried gills. This was used to determine morphological differences between the dried materials, which could be used to classify the dried specimens into species, without the use of DNA data when conducting management or enforcement.

Visual morphological differences of dried ray gill rakers were subsequently obtained, and classification errors of gill rakers characteristics in each species were tested. We consequently presented a rapid practical identification method based on macroscopic morphology of specimens. This method is particularly beneficial for management and enforcement officers as well as for researchers who intend to study the biodiversity conservation of *Manta* rays in both source and consumer countries. Some recommendations for the global protection of *Manta* and *Mobula* rays were also provided in the article. If the *Mobula* rays were to be included in the CITES Appendices, we also hope that our work can be of assistance in identification and implementation of CITES for these species as well.

## Results

### Species identification and market component

Not all specimens were successfully amplified because of the low DNA quality in some dried gills. In this study we successfully sequenced 761 bp of the COI gene and 1033 bp of the NADH2 gene resulting in 35 and 58 haplotypes respectively. All of the haplotype sequences generated were deposited in GenBank under the accession numbers KT626464-KT626550. Based on DNA barcodes of COI and NADH2 genes, 188 individuals were successfully identified to the corresponding species with 99–100% identical values compared with those deposited sequences in NCBI. Five monophyletic clusters of individuals (with 99–100% supports) were recovered in Neighbor-joining (NJ) trees under the Kimura 2-parameter (K2P) model in both genes, corresponding to five species of Mobulid rays (Appendix I and II). Although the detail calculation was not shown in the tables, the intra species distances were 0–1.1% in COI gene and 0.3–0.6% in NADH2 gene, while those inter species distances were 3.7–12.5% and 4.4–17.1%, respectively. Finally five species of rays were identified (Table 1), namely, *Mobula japanica* (Müller & Henle, 1841), *Mobula tarapacana* (Philippi, 1892), *Mobula kuhlii* (Müller & Henle, 1841), *Mobula thurstoni* (Lloyd, 1908) and *Manta birostris* (Walbaum 1792), representing 54.8%, 14.4%, 13.3, 6.4% and 11.2% of the sample set, respectively (Table 1).

### The price changes among various species or in different localities

**Figure 1:**
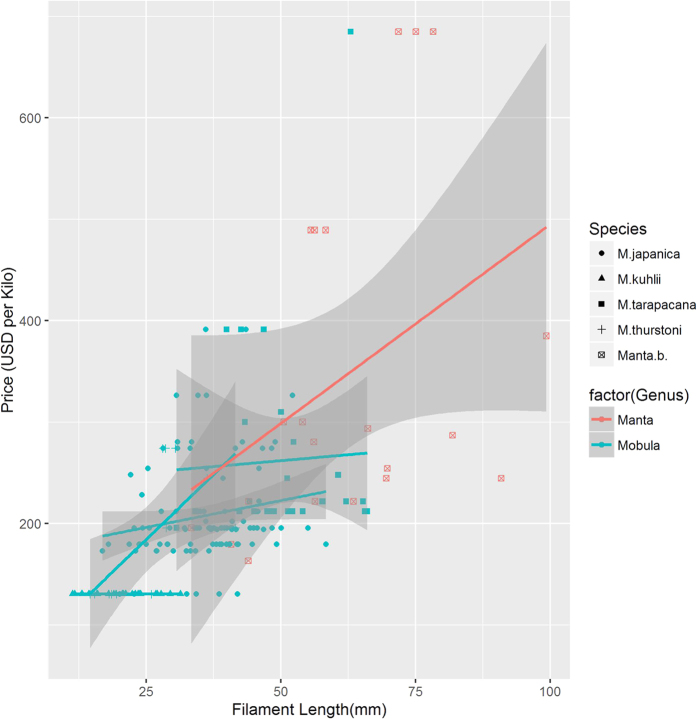
Price trends for dried gill rakers in South China markets, according to size.

Dried gills of five species were sold at an average price of 220 ± 98 US$ per kilogram in China (Table 1). Regardless of whether it is loose or tight (F = 0.150, P = 0.861) or in any colors (F = 0.566, P = 0.638), a dried ray gill is likely more expensive when the average length of filaments is long (F = 19.486, P < 0.01). Excluding the influence of length of the gill filaments on price, the prices of dried ray gill rakers are significantly different among sampling cities (F = 30.188, P < 0.01). The average price of dried gill rakers were 201 ± 82 US$ in Guangzhou, 229 ± 47 US$ in Zhanjiang, 290 ± 25 US$ in Beihai, 294 US$ in Zhapo and 445 ± 159 US$ in Shanwei, respectively. Regardless of the abovementioned influences, the price of dried gill rakers of *Manta* rays is significantly different to that of *Mobula* rays (AIC = 2773.8, t = 8.986, P < 0.01, Fig. 1).

### Visual discriminations and identification

**Figure 2:**
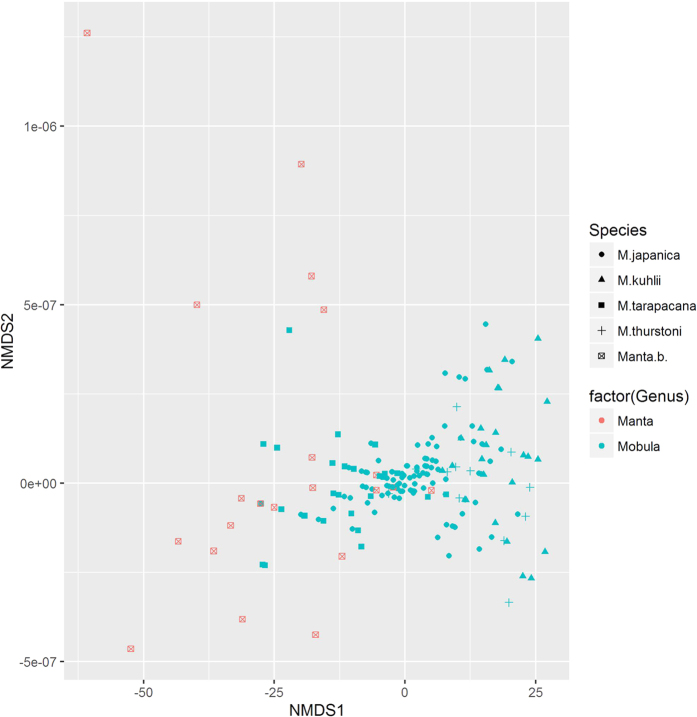
Multidimensional scaling 2-dimensional visualization of sizes of dried ray gills in South China markets.

Tukey’s multiple comparisons of the mean filament length of each species revealed that the filament lengths of the dried gill of each species are significantly different from one another (P < 0.01), except for the differentiation between *Mo. thurstoni* and *Mo. kuhlii* (P = 0.54) (Table 2). Gill rakers of *Ma. birostris* have lengths ranging from about 30 mm to almost 100 mm, and those longer than 70 mm are usually from this species (Fig. 1). Specimens however, cannot be distinguished into species only based on the filament lengths or sizes of the gill rakers (Fig. 2).

Unlike the dried gill rakers of other *Mobula* rays, the specimens of *Mo. tarapacana* and *Manta birostris* can be reliably distinguished via the visual method (Table 2). The dried gill rakers of *Ma. birostris* and *Mo. tarapacana are* tight arranged with large-terminal lobes that are joined together. Finger-like projections were observed at the lower edge of each lobe in *Manta* species, but these projection structures are invisible in several small or poorly preserved specimens. The dried gill rakers of *Mo. tarapacana* are mostly dark in color with a light base and the so-called variegated gills (Hua Sai in Chinese). Moreover, cilia can be seen under a hand loupe in some well-preserved specimens.

Most terminal lobes of dried gill of *Mo. japanica* have a sharp apex and are independent from each other, and thus the lobes could be used in species discrimination. *Mo. kuhlii* and *Mo. thurstoni* both have oval-shaped gill lobes and their gill rakers are similar in size; thus, discriminating the two species from each other is difficult (Table 2). Overall, when all types of visual traits are used in the random forests formula, most specimens of dried gill rakers could be discriminated to the DNA-identified species level with classification errors of 3.88% in *Mo. japanica*, less than 0.1% in *Ma. birostris* and *Mo. tarapacana*, 16% in *Mo. kuhlii and* more than 99.9% in *Mo. thurstoni* (Table 2). A total estimate of error rate is 10.64%. The characteristics of middle lobes are the most useful trait in visual classification followed by the characteristics of the terminal lobes.

**Figure 3:**
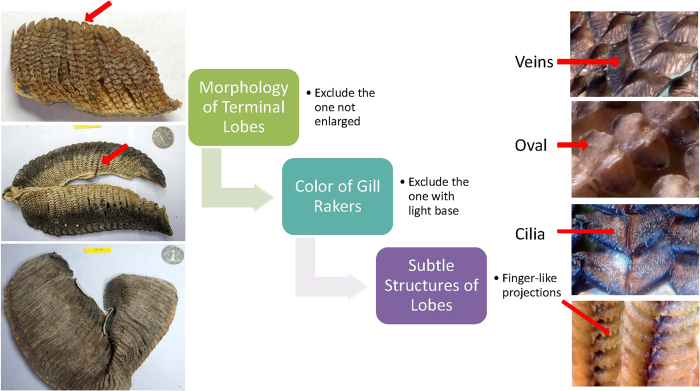
A three-step process for identifying the gill rakers of manta rays.

For frontline enforcement at customs or border inspection, we develop a three-step process for identifying the gill rakers of *Manta* rays. The process involves weeding out specimens at the first step, inspecting whether or not terminal lobes are enlarged and fused. The variegated gills are then abandoned, and the finger-like projections are finally observed at the edge of middle lobes through a loupe (Fig. 3).

## Discussion

The fishing of Mobulids for their dried gill rakers had not been given particular attention until the turn of the century^7^,^18^. *Ma. birostris* was listed on Appendices I and II of the Convention on Migratory Species (CMS) in 2011. Currently, increasing concern has been given to this activity and culminated in an adoption of the proposal to amend CITES Appendix II in March, 2013. In early 2014, Indonesia, which was reported to be the largest landing country of *Manta* rays^19^, has declared a ban on fishing *Manta* rays^20^,^21^. All international treaties need effective local and national implementation and national fisheries management within relevant parties^14^. The synergies among different treaties rely on the parties’ willingness and capacity of mainstreaming the administrative and enforcement power of its ministries and sectors, as well as those in the provinces. If so done, it is believed that the increasing threat of gill rakers trade will be curbed by following the reinforcement of international treaties, implementation and enforcement of national laws and regulations, regulation of the demand of side markets, regional cooperation, and national fishery management to ensure the survival of *Manta* rays, as well as *Mobula* rays. As a follow-up to the solution to the identification of *Manta*s, it is therefore imperative that further field studies are carried out around the world to quantify the extent and impact of *Manta* fishery and trade and to monitor the products flow in order to ensure the preservation of *Manta* and *Mobula* ray species and populations worldwide.

In this study, five species, *Mobula japanica, Mo. tarapacana, Mo. kuhlii, Mo. thurstoni*, and *Manta birostris*, have been firstly verified to be traded within China. The taxonomy of the genus *Manta* has been questionable and convoluted, having previously been considered a monotypic genus possibly with three species *Manta birostris, Ma. alfredi* and *Ma. sp. cf. birostris*, but further examination of specimens is necessary to clarify the taxonomic status of Manta species^11^. Aslo, *Manta alfredi* and *Manta birostris* are indistinguishable based on both the CO1 and NADH2 genes^22^,^23^. Much more sensitive molecular marker should be found and used in the further study because accurate species identification will help to highlight the specific threats and will ultimately assist in the correct assessment of the conservation status in each different species of *Manta*. Only *Mobula japanica* and *Mo. tarapacana* could be distinguished from other *Mobula* species by the results of this study (Table 2). The morphological approach of gill rakers does not appear robust to distinguish the remaining *Mobula* rays at the present stage. Given that Mobula are likely as threatened as Manta, and are also slated for inclusion under CITES in October, 2016, we continue doing some works on the morphology of the surface of gill lamellae by using SEM. However, for conservation management and customs enforcement, establishing a kind of voucher sequences for each Mobulid species that can be used as the baseline for comparison moving forward is urgent needed. Ideally, these will be tied to voucher specimens, or alternatively, sequences linked to photographs of whole specimens and gill raker, morphological diagnosis characteristics, as well as meristic data. Such approach is also applicable to all CITES listing species, and need the great effort and collaboration between source countries and consumed counties.

In the IUCN Red List of Threatened Species, *Ma. birostris* and *Ma. alfredi* (Krefft, 1868) are listed as “Vulnerable”^24^ and the *Mobula* rays involved in this research are all listed as “Near Threatened” or “Data Deficient”^25^,^26^,^27^,^28^. The population trends of *Ma. birostris, Ma. alfredi* and *Mo. kuhlii* are decreasing^24^,^25^, but those of other species are unknown. Mobulids may have a similar fecundity rate, where one adult female gives birth to one pup annually. However, the life history parameters of four *Mobula* species in the present research, namely, age at maturity, longevity, reproductive age, and natural mortality, and even population genetics are mostly unknown^6^,^26^,^27^,^28^. Thus, scientists and fishery organizations must promote biological, ecological and molecular studies on Mobulid rays, especially on those species largely used for their gill rakers, such as *Mo. japanica*.

*Mo. kuhlii* are found in the Indian Ocean and in the Western Central Pacific. On the other hand, *Manta birostris, Mo. japanica, Mo. tarapacana*, and *Mo. thurstoni* are circumglobally found in tropical to temperate seas. Compared with the five species involved in this research, *Manta alfredi* and the rest of the Mobula rays are mainly regionally distributed in the Eastern Pacific, Atlantic, and Mediterranean Sea and in surrounding waters^4^,^6^. Moreover, the diver project database reported that Mobulids are fished and then sold in local markets in Sri Lanka, Indonesia, and East Africa^16^. Therefore, it is presumed that dried gill rakers in Chinese markets are mainly from rays fished and traded from the Indian Ocean and the Western Central Pacific. The trade in dried gill rakers in China may affect coastal countries around the Indian Ocean and Western Central Pacific, which have been identified as one of the main hotspots where sharks and rays are seriously threatened^2^. More analyses are urgently needed on the genetic backgrounds of local populations, on tracing the origin of *Ma. birostr*is, location of harvests, populations size, and distribution and trends from the harvest sites to determine key resource countries that would receive international assistance and cooperation for sustainable fishery management, implementation of international treaties, and enforcement measures.

From the size distribution and the morphological characters of dry gill raker, 2 empirical rules can be presented for enforcement officers. The results of filaments size and price investigation reveal the filament lengths of *Ma. birostris* to be from about 30 mm to almost 100 mm with an average of 62.6 mm (Fig. 1), showing a largest mean value and a widest distribution among all species and indicating a variety of sizes of this species consumed for its gill rakers. The gill rakers of *Ma. birostr*is are also generally larger than those of *Mobula* rays (Fig. 1). Among all species of *Mobula* rays, adult fish rakers of *Mo. tarapacana* are large and can even rival *Manta* rays in size^5^. Although some of the rakers of *Ma. birostris* are similar in size compared with those of *Mo. tarapacana*, many specimens from *Ma. birostr*is are significantly larger than those of *Mo. tarapacana*. The filaments longer than 70 mm are usually from *Ma. birostris* (Fig. 1). Hence, a larger size of gill rakers whose filaments are longer than 70 mm would be an important criterion for enforcement officers. Based on the results of this study, we also develop a three-step process for identifying the gill rakers of Mobulid rays which can be result in a classification errors of 3.88% in *Mo. japanica*, and less than 0.1% in *Ma. birostris* and *Mo. Tarapacana* (Table 2, Fig. 3). These three species have higher commercial value and occupy more than 80% of the market component (Table 1). The three-step process of identifying gill rakers of *Manta* rays has already been trained and greatly improved the efficiency of law enforcement of frontline inspections, customs, and fishery management administrations in China.

The analyses on market components firstly show that the price of dried gill rakers of *Ma. birostris* is significantly different with that of Mobula rays, even if the influence of the filament size on the price is removed (Fig. 1). Our research revealed that markets of ray gill rakers in China have changed in the past few years. In 2011, small gill rakers were priced at US$133 per kg. Each kilogram of large gill rakers was sold at a high price of up to US$500^9^. Our study showed that the recent price of small gill rakers has not distinctly changed, and the highest price per kilogram of large gill rakers mainly from *Ma. birostris* has increased to about US$685. At the same time, the occupancy rate of dried gill rakers from *Manta* rays decreased from around 30%^8^ to 11.2%. The trade of *Manta* rays would be affected by the adoption of a proposal to amend CITES Appendix II, although the list was not yet enforced at the time of sampling. Thus, the change in the markets of gill rakers may be a result of a series of national, regional, and international measures recently issued or, a bold assumption of possible resource exhaustion. Close attention should be paid to the subsequent trend of price and percentage composition of *Manta* gill rakers in the near in future.

Based on our investigation, consumption of gill rakers in Mainland China is mainly limited to two southern provinces, namely, Guangdong and Guangxi. The vast majority of the markets are in Guangzhou. The consumption of wildlife as food in Guangzhou is mostly driven by utilitarian motivation, but consumers prefer “selective protection” and “protected according to the law”, which means they are more likely to refuse to consume a product of wildlife if it is listed to be protected by the law^29^. Thus, the trade regulation and restrictions on ray gill rakers to businessmen must be enforced to facilitate the regulation and to promote conservation strategies on markets to consumers. Moreover, the public must be made aware of the biological and ecological values of Manta and Mobula rays, and the regulation and law enforcement on market should be strengthened. For a long time, landed Mobulids have mainly been used as fish meal in China^30^. Gill or other parts of Mobulids have never been recorded in the Chinese Pharmacopoeia or a classical literature in the past dynasties of China^31^,^32^. Only in specific communities in costal Guangdong Province are gill rakers known as folk medicine and are taken for helping to relieve exterior syndrome by diaphoresis or children measles and skin sores and boils1^5^,^32^. Demand reduction is widely recommended for resolving the challenges that endangered species threatened by massive trade. In this situation, to reduce nonessential demand for gill rakers from Mobulids, we suggest promoting medical science popularization about related diseases but also protection awareness. These strategies will help control the demand of side markets.

Overall, in this study, 83% of sampled specimens of dried ray gill rakers were successfully identified to species level, and five species of Mobulid rays were discovered. Gill of *Manta birostris* occupies a relatively smaller part of market component and was sold at a higher price. As a DNA barcodes and morphology-related biodiversity conservation approach, we develop a rapid and accurate species identification method to address the uncertainties in identifying dried gill rakers of *Manta* and *Mobula* rays. We hope that our work can serve as a foundation and basis to reinforce objectives of international treaties, regulation of consumer-driven markets, regional cooperation, and national fishery management to curb the increasing threat of gill rakers trade to ensure the survival of *Manta* and *Mobula* rays. If the *Mobula* rays were to be included in the CITES Appendices, we also hope that our work can be of assistance in identification and implementation of CITES for these species as well.

## Methods

### Sampling

Given that dried gill rakers of *Manta* and *Mobula* rays have been only consumed in southern China, sampling was performed in fish markets and dry seafood markets in five cities in Guangdong province and Guangxi Zhuang Autonomous Region, from November 2013 to August 2014. The samples were randomly collected from each booth but with full consideration of size, coloration, price (recorded in US$ per kilogram), as well as the intervals between adjacent gill rakers. A total of 224 samples were collected and kept in dry conditions for DNA extraction and morphological measurement.

### Species-level molecular identification

Whole-genomic DNA was extracted using the TIANamp Genomic DNA Kit (TIANGEN BioSci. and Tech. Co., Beijing, China) following the manufacturer’s recommendations and assessed with 1.0% agarose gel. The mitochondrial cytochrome oxidase subunit I (COI) and NADH2 gene analyses are currently the most effective strategies for species identification and recognition in elasmobranches (589 and 574 of the 1221 recognized elasmobranch species were recorded by COI in FISHBOL and by NADH2 in NCBI, respectively)^22^, which provide an appropriate genetic resource for comparison and for finding similarities between our results and deposited ones. Thus, we chose the double molecular markers for species identification. Two dedicated primers for each gene were designed using OLIGO 6^33^.

COI-F0: TAACTCTCAGCCATCTTACC,

COI-R823: GCCGATAGCTATTATTGCTCA,

ND2-F2: GCCCATACCCCAACCATGTT and

ND2-R2: TGCCTATCTAGAAGGTTTTAGCT

DNA amplification by PCR was carried out in a final volume of 30 μL, containing 15 μL of 2 × Taq PCR MasterMix, 1 μL of each primer, 1 μL to 2 μL genomic DNA, and ddH2O. The PCR thermocycling conditions for COI gene were 95 °C for 5 min, followed by 35 cycles of 95 °C for 30 s, 52 °C for 30 s, 72 °C for 50 s, and a final extension of 72 °C for 10 min. The conditions for the ND2 gene were the same as those for the COI gene, except that the annealing temperature was 54 °C. The PCR amplification products were sequenced at Sino Geno Max (Beijing) with the forward and reverse primers used for the amplification. Sequences were assembled and edited using CLUSTAL W^34^ and DNAStar 7.10 ((DNASTAR Inc.). Consensus sequences were blasted through NCBI or BOLD web-based systems. The hits with the highest coverage and maximum identical values were chosen to assign an unknown sample to a known species. Furthermore, a neighbour-joining analysis was used to explore sequence differences at inter or intra-species level.

### Morphological measurements

**Figure 4:**
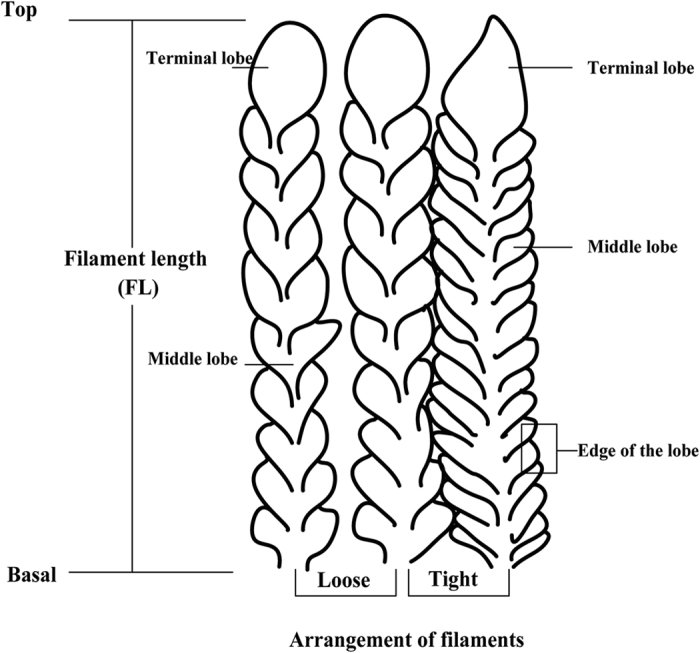
Diagram of part of gill raker of Mobulid ray, showing morphology and arrangement of gill filaments and secondary lamellae.

A small number of museum collections revealed that the filter lobe morphology and ultrastructures of Mobulid rays can be used as a key diagnostic parameter among some species^5^,^35^. For each filament plate of gill rakers, the filament lengths were measured (Fig. 4, FL, mm) using a digital vernier caliper at five points: two from edges of the gill raker plate, one from the midpoint, and two between the edge and the midpoint. The average was calculated as the filament length and was used in further analyses.

Based on the combined information of the filament and lobe morphology^5^,^28^, four diagnostic characteristics, namely, arrangements and colors of filaments, shapes or structures of middle lobes, and shapes of terminal lobes, were chosen to evaluate the discriminating availabilities of each species. For the arrangement of filaments, three levels were used for intuitionistic observation, namely, loose, general, and tight. A loose arrangement means that each filament has clearance at the edge. A tight arrangement means that the filaments almost overlap at the edge with adjacent ones, and the general arrangement is the midpoint of a loose and tight arrangement (Figs 3 and 4).

The samples included four kinds of independent traits in the middle lobes of dried gill rakers, which are three kinds of subtle structures viewed through a 30X-21 mm magnifying loupe and an oval lobe shape. Finger-like projections are macroscopic dentils extending down from the ventral edge of a lobe, and the lobe surface is smooth in appearance. Cilia are tiny hairs on the surface of the lobe, and veins are dark, thin, vein-like lines on the lobe surface. Except for the three abovementioned subtle structures and the oval shape, several specimens have no remarkable characteristic on the middle lobes (Figs 3 and 4).

Three types of shape of terminal lobes could be used in species identification. Several specimens have enlarged lobes on the terminal end where lobes are joined or fused together. In several specimens, the apex of the lobes is sharp and does not come into contact with the terminal lobes of adjacent filaments. The third kind of terminal lobe has a similar size with the middle lobes and also has an oval shape.

### Analysis and statistics

R version 3.3.1 was used in statistical analysis and figure constructions. Shapiro–Wilk test was performed in the normality tests of data. One-way ANOVA was performed to compare the differences in price of gill rakers among three types of arrangement among different colors and among cities. We also used a general linear model to analyze the relationship between the price and the size of dried gill rakers, treating species, site, arrangement, and color as cofactors. Tukey’s multiple comparisons were performed to test the differentiations among the filament lengths of each species identified via DNA barcoding. We used Breiman and Cutler’s random forests for classification and regression^36^,^37^ based on a forest of 1000 trees by using random inputs in testing the availability of each characteristic in visual discrimination. Multidimensional scaling is used to visualize the dissimilarities among specimens based on their sizes^38^.

## Additional Information

**How to cite this article**: Zeng, Y. *et al*. DNA barcoding of Mobulid Ray Gill Rakers for Implementing CITES on Elasmobranch in China. *Sci. Rep.* **6**, 37567; doi: 10.1038/srep37567 (2016).

**Publisher's note:** Springer Nature remains neutral with regard to jurisdictional claims in published maps and institutional affiliations.

## Supplementary Material

Supplementary Information

## Figures and Tables

**Table 1:** Species Components of Ray Gill Rakers in Mainland China Markets.

	*Mo. japanica*	*Mo. kuhlii*	*Mo. tarapacana*	*Mo. thurstoni*	*Manta birostris*
Individual number	103	25	27	12	21
Percentage	54.80%	13.30%	14.40%	6.40%	11.20%
Average price (US$/Kilogram)	208 ± 50	130	267 ± 103	187 ± 64	348 ± 170

**Table 2:** Variability of characteristics of dried gill rakers for visual discrimination.

		*Manta birostris*	*Mo. japanica*	*Mo. tarapacana*	*Mo. kuhlii*	*Mo. thurstoni*	Estimates of error rate
Mean filament length (mm)		62.6 ± 16.7	36.0 ± 8.4	51.4 ± 9.8	20.4 ± 5.7	25.5 ± 8.5	43.62%
Arrangement of filaments	Loose	0	59%	0%	64%	50%	38.83%
	General	48%	33%	26%	36%	50%	
	Tight	52%	8%	74%	0	0%	
Color of filaments	Light end	0	30%	0	0	0	31.38%
	Light base	5%	2%	93%	44%	58%	
	Whole light	33%	4%	4%	4%	0	
	Whole dark	62%	64%	4%	52%	42%	
Characteristics of middle lobes	Finger-like projections	81%	0	0	0	0	13.30%
	Veins	0	52%	0	8%	0	
	Oval	0	2%	0	92%	100%	
	Cilia	0	0%	81%	0	0	
	No obvious characteristics	19%	46%	19%	0	0	
Characteristics of terminal lobes	Large even jointed	100%	2%	100%	0	8%	22.87%
	Oval	0	2%	0	76%	67%	
	Sharp apex	0	96%	0	16%	8%	
Classification error		<0.1%	3.88%	<0.1%	16%	>99.9%	
