# Supplementary material for: DNA barcoding of Mobulid Ray Gill Rakers for Implementing CITES on Elasmobranch in China
Source: Sci Rep. 2016 Nov 23;6:37567. doi: 10.1038/srep37567 (PMC5120345; doi:10.1038/srep37567)

**DNA barcoding of Mobulid Ray Gill Rakers for Implementing CITES on Elasmobranch in China**

Yan Zeng^1,2^, Zhongze Wu^3^, Chunguang Zhang^1^, Zhibin Meng^1,2^, Zhigang Jiang^1,2^ and Jie Zhang^1*^

^1^ Institute of Zoology, Chinese Academy of Sciences, Beijing 100101, China.

^2^ Endangered Species Scientific Commission, People’s Republic of China, Beijing 100101, China.

^3^ CITES Management Authority, People’s Republic of China, Beijing, 100714, China

*Corresponding author: Jie Zhang,

Institute of Zoology, Chinese Academy of Sciences.

1 Beichen West Road, Chaoyang District, Beijing 100101, P. R. China.

Tel: +86-10-64807076; Fax: +86-10-64807099,

E-mail address: [zhangjie@ioz.ac.cn](mailto:zhangjie@ioz.ac.cn)

**Supplementary information**

1. Appendix I. Neighbor-joining tree constructed with COI haplotypes of Mobulid rays.
2. Appendix II. Neighbor-joining tree constructed with NADH2 haplotypes of Mobulid rays.


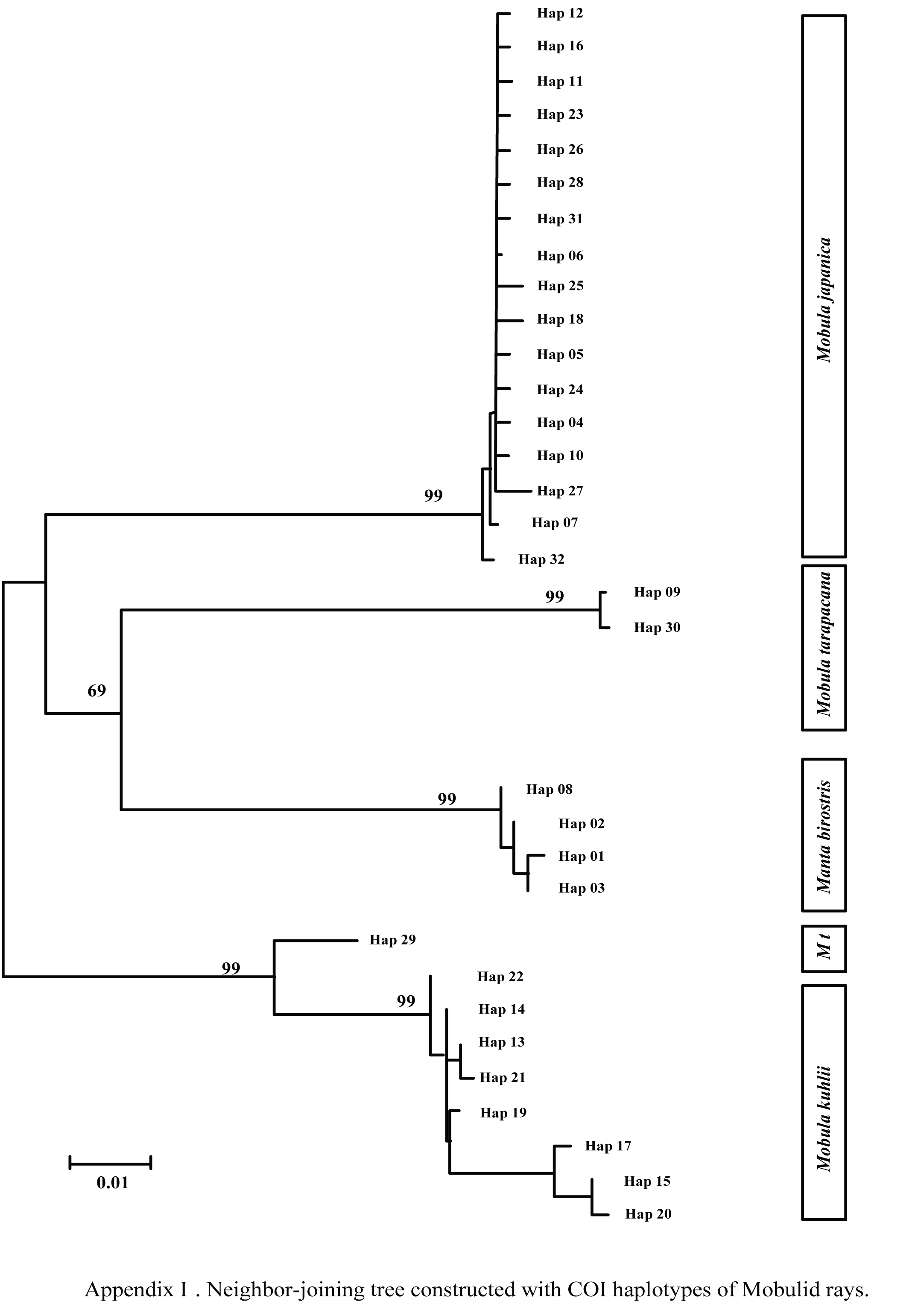

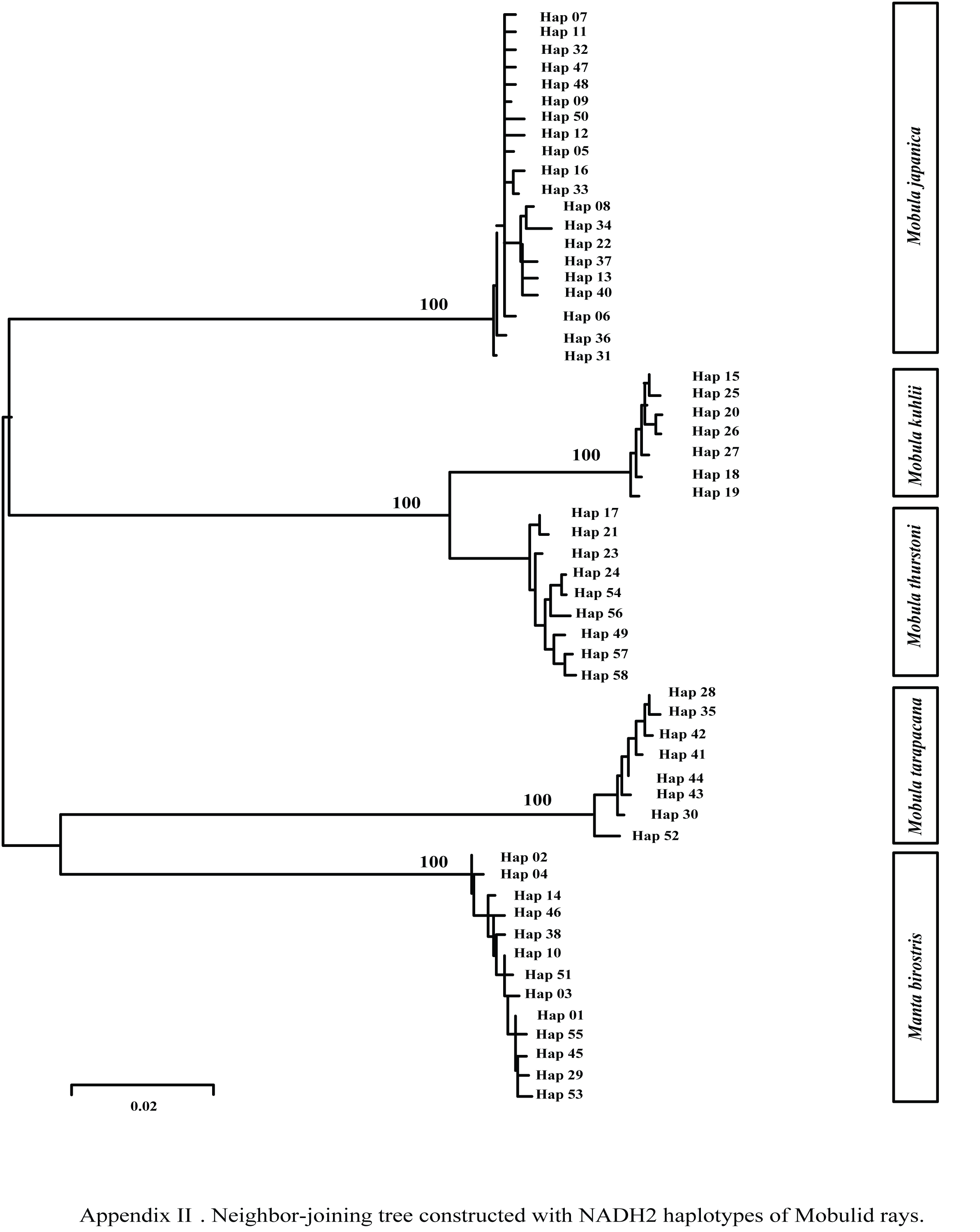

Supplement: Supplementary Information [file srep37567-s1.doc]
